# Whole-Genome-Based Web Genomic Resource for Water Buffalo (*Bubalus bubalis*)

**DOI:** 10.3389/fgene.2022.809741

**Published:** 2022-04-11

**Authors:** Aamir Khan, Kalpana Singh, Sarika Jaiswal, Mustafa Raza, Rahul Singh Jasrotia, Animesh Kumar, Anoop Kishor Singh Gurjar, Juli Kumari, Varij Nayan, Mir Asif Iquebal, U. B. Angadi, Anil Rai, Tirtha Kumar Datta, Dinesh Kumar

**Affiliations:** ^1^ Centre for Agricultural Bioinformatics, ICAR-Indian Agricultural Statistics Research Institute, New Delhi, India; ^2^ ICAR-Central Institute for Research on Buffaloes, Hisar, India

**Keywords:** bovine, lncRNA, miRNA, molecular markers, web-resource, CircRNAs

## Abstract

Water buffalo (*Bubalus bubalis*), belonging to the *Bovidae* family, is an economically important animal as it is the major source of milk, meat, and drought in numerous countries. It is mainly distributed in tropical and subtropical regions with a global population of approximately 202 million. The advent of low cost and rapid sequencing technologies has opened a new vista for global buffalo researchers. In this study, we utilized the genomic data of five commercially important buffalo breeds, distributed globally, *namely*, Mediterranean, Egyptian, Bangladesh, Jaffrarabadi, and Murrah. Since there is no whole-genome sequence analysis of these five distinct buffalo breeds, which represent a highly diverse ecosystem, we made an attempt for the same. We report the first comprehensive, holistic, and user-friendly web genomic resource of buffalo (*BuffGR*) accessible at http://backlin.cabgrid.res.in/buffgr/, that catalogues 6028881 SNPs and 613403 InDels extracted from a set of 31 buffalo tissues. We found a total of 7727122 SNPs and 634124 InDels distributed in four breeds of buffalo (Murrah, Bangladesh, Jaffarabadi, and Egyptian) with reference to the Mediterranean breed. It also houses 4504691 SSR markers from all the breeds along with 1458 unique circRNAs, 37712 lncRNAs, and 938 miRNAs. This comprehensive web resource can be widely used by buffalo researchers across the globe for use of markers in marker trait association, genetic diversity among the different breeds of buffalo, use of ncRNAs as regulatory molecules, post-transcriptional regulations, and role in various diseases/stresses. These SNPs and InDelscan also be used as biomarkers to address adulteration and traceability. This resource can also be useful in buffalo improvement programs and disease/breed management.

## Introduction

Water buffalo, scientifically known as *Bubalus bubalis,* is the major source of milk, meat, and drought in various countries, making it an economically important animal. This livestock species belonging to the *Bovidae* family is mainly distributed in tropical and subtropical regions. Based on morphology and behavior, the two categories of domestic Asian water buffalo are river buffalo (2n = 50) and swamp buffalo (2n = 48) (Iannuzzi, 1994). The global population of water buffalo is ∼217 million in 34 countries (FAOSTAT, 2020) with ∼82% and ∼18% river buffalo and swamp buffalo, respectively. South Asia holds the majority of water buffalo as India ranks first in buffalo breeding with a share of 50.5%, followed by Pakistan and China (Taşcioğlu et al., 2020). Buffalo are largely domesticated by small farmers in Asia. This indicates the popularity and dependence of water buffalo as compared to any other species that are domesticated. India has the lion’s share (69%) in river buffalo. Milk yield is more in buffalo than cattle. Also, buffalo milk has a higher nutritional value than cattle on account of higher fat content (8.0%), higher unsaturated fatty acid levels, higher protein content (4.5%), and lower phospholipid and cholesterol levels. It is a more preferred milk for dairy products (Du et al., 2019).

The fast decline in DNA sequencing costs has paved the way for researchers across the globe to revolutionize genome analysis. Assembly and decoding of the genome of water buffalo is in continuous progress. The following five genome assemblies of water buffalo exist on the NCBI database (https://www.ncbi.nlm.nih.gov/assembly/?term=Bubalus) (Last accessed: July 2021): GCA_003121395.1 of the Mediterranean breed from University of Adelaide, Australia; GCA_019923935.1 of the Murrah breed from National Dairy Development Board, India; GCA_004794615.1 of the Bangladesh breed from BGI-Shenzhen, China; GCA_002993835.1 of the Egyptian buffalo breed from Agriculture Genetic Engineering Research Institute and Nile University, Egypt, and GCA_000180995.3 of the Jafarabadi breed from Anand Agricultural University, India. GCA_003121395.1 and GCA_019923935.1 are chromosome level assemblies with 25 chromosomes, however, RefSeq annotation has not yet been provided for GCA_019923935.1.

Whole-genome sequencing and transcriptome studies provide insights on genetic makeup, numerous trait markers, and their expression in organisms. Simple sequence repeats (SSR) are the information source for genetic diversity among different breeds/varieties of the same species (Patzak et al., 2012). There are 22 buffalo breeds (only river subspecies) distributed all over the world with different characteristics like shape, size, color, weight, and lactation period, etc. Genomic variation results in single nucleotide polymorphisms (SNPs), insertions, and deletions (Surya et al., 2018). These variations are stable and are transferred from one generation to the next. These variations impinge start codon gain or loss, stop codon gain or loss, or frame shift. The presence of such variations in protein coding regions culminates in synonymous or non-synonymous amino acid replacement.

Long non-coding RNAs are a group of RNAs which are greater than 200 nt and lack open reading frames or have <100 amino acids in length. lncRNAs regulate gene expression through methylation and demethylation (Bhat and Jones 2016; Fernandes et al., 2019) and through chromatin modifications by interfering with transcription factors [binding with DNA and regulating transcription (Griffiths et al., 2000)] and miRNAs. lncRNAs perform post-translational regulation through capping, alternative splicing, editing, transport, translation, degradation, and stability of mRNA targets. Apart from their biological roles, lncRNAs can also function as biomarkers. At the organism level, lncRNAs are known to be abnormally expressed in many diseases therefore playing a role in diagnosis (Kosinska-Selbi et al., 2020).

miRNAs are 18–25 nucleotide-long regulatory sequences, which play an important role in response reactions during anorganism’s exposure to biotic or abiotic conditions (O’Brien et al., 2018). They regulate gene expression by binding to the target sequence with the help of AGO protein and make an miRNA-induced silencing complex (mi-RISC) (Kawamata and Tomari, 2010). Water buffalo are adapted to higher to lower altitudes, hence they face a wide range of stresses like low/high temperatures (Liu et al., 2019), pathogens (Dhanoa et al., 2019; Lecchi et al., 2019), etc. Previously known miRNAs specific to buffalo have been reported from various transcriptome studies involving such stress conditions (Dhanoa et al., 2019; Lecchi et al., 2019; Liu et al., 2019).

Other regulatory non-coding RNAs, known as circular RNAs (circRNAs), spawn through back-splicing of RNAs. They are more stable than RNAs (Chen et al., 2017; Wang et al., 2017). The functions of circRNAs are not well known but still it is reported that they play a significant role in post-transcriptional regulation of gene expression (Lukiw, 2013). CircRNAs function as a sponge of miRNAs by sequestering them by binding and interacting with lncRNAs (Lei et al., 2021). These are being employed as biomarkers for controlling and treating diseases (Meng et al., 2017; Lu, 2020).

Before release of the buffalo reference genome, most of the studies related to buffalo involving omics analyses were based on the *Bos taurus* reference genome. The available whole-genome assemblies of five buffalo breeds represent a highly diverse ecosystem. Their utilization in whole-genome sequence analyses and in extraction of rapid polymorphic markers at lower costs for the breeders is warranted. In 2018, a buffalo reference genome with 24 chromosomes along with X and MT chromosomes was released by the Italian Buffalo Genome Consortium (https://www.ncbi.nlm.nih.gov/assembly/GCA_003121395.1). For the current study, the different omics studies in buffalo were performed using the GCA_003121395.1 buffalo reference genome to extract non-coding RNAs such as miRNAs, lncRNAs, and circRNAs in the 31 buffalo tissues, which had not been attempted earlier. Also, the various genetic markers such as SSRs, SNPs, and InDels from five breeds of buffalo (Mediterranean, Egyptian, Bangladesh, Jaffrarabadi and Murrah) were mined. After extraction of the mentioned molecular markers and non-coding RNAs, a web-based genomic resource, *BuffGR* was developed to facilitate the buffalo research community with user-friendly, single-window retrieval of buffalo *omics* data to be utilized for further scientific research and studies. This buffalo web resource is state-of-the-art, holistic, and currently the largest collection related to buffalo including the most important breed of India, i.e., Murrah from the latest 2021 assembly as well as the world, i.e., Mediterranean from the latest 2018 assembly.

## Materials and Methods

### Data Retrieval and Processing

In order to extract the breed-wise molecular markers and variants like SSRs, SNPs, and InDels, in five buffalo breeds, *namely*, Mediterranean, Egyptian, Bangladesh, Jaffrabadi, and Murrah, their genome assemblies were retrieved from NCBI (Table 1).

**TABLE 1:** The list of assemblies of buffalo from public domain.

Accession	Breed	Submitter	Assembly level	Remarks
GCA_003121395.1	Mediterranean	University of Adelaide	Chromosome-wise	UOA_WB_1 (https://www.ncbi.nlm.nih.gov/assembly/GCF_003121395.1/)
GCA_019923935.1	Murrah	National Dairy Development Board, India	Chromosome-wise	NDDB_SH_1 (https://www.ncbi.nlm.nih.gov/assembly/GCF_019923935.1/)
GCA_004794615.1	Bangladesh	BGI-Shenzhen	Scaffold level	Bubbub1.0 (https://www.ncbi.nlm.nih.gov/assembly/GCA_004794615.1/)
GCA_002993835.1	Egyptian	Egyptian Water Buffalo Genome Consortium (Agriculture Genetic Engineering Research Institute and Nile University)	Scaffold level	ASM299383v1 (https://www.ncbi.nlm.nih.gov/assembly/GCA_002993835.1/)
GCA_000180995.3	Jaffrabadi	Anand Agricultural University, Anand, Gujarat, India	Scaffold level	Bubalus_bubalis_Jaffrabadi_v3.0 (https://www.ncbi.nlm.nih.gov/assembly/GCA_000180995.3/)

For extraction of cirRNAs, SNPs, and InDels, RNA-seq data of a total of 31 buffalo tissues were retrieved from NCBI, which were mapped with the GCF_003121435.1 genome assembly of the Mediterranean breed using Bowtie2 (Langmead and Salzberg, 2012), while HISAT2 (Kim et al., 2019) was used in the case of lncRNAs (Table 2). For the extraction of miRNAs, cirRNAs, and lncRNAs, the genome assembly of the Mediterranean breed was used (GCF_003121435.1).

**TABLE 2:** The details of RNA-seq data from the International Water Buffalo Genome Project representing different buffalo tissues along with SRA IDs and mapping %.

Tissue	SRA IDs	Mapping %	Tissue	SRA IDs	Mapping %
Tongue	ERR315616	95.71	Ovary-corpus luteum	ERR315632	94.30
Rumen	ERR315617	93.69	Ovary follicle	ERR315633	97.60
Abomasum	ERR315618	95.91	Oviduct	ERR315634	96.67
Small intestine	ERR315619	93.88	Endometrium	ERR315635	96.59
Large intestine	ERR315620	96.03	Mammary gland	ERR315636	95.18
Obex	ERR315621	94.02	Embryo pool	ERR315637	70.87
Hypophysis	ERR315622	96.84	Embryo single	ERR315638	73.61
Spinal Cord	ERR315623	95.40	Thymus	ERR315639	96.71
WBC	ERR315624	97.04	Mesenteric lymph node	ERR315640	96.47
Cerebellum	ERR315625	90.61	Spleen	ERR315641	96.07
Bone Marrow	ERR315626	95.55	Liver	ERR315642	96.57
Muscle longissimus dorsai	ERR315627	96.21	Pancreas	ERR315643	96.70
Muscle semitendinosus	ERR315628	96.62	Kidney	ERR315644	95.23
Testis	ERR315629	97.40	Lung	ERR315645	96.53
Thyroid	ERR315630	96.19	Testis	SRR527266-72	90.02
Heart	ERR315631	94.68	Milk	SRR7091387-98	94.88

### Identification of SNPs and InDels

For extraction of variants, *namely,* SNPs and InDels, the four buffalo breeds (Murrah, Jaffrabadi, Bangladesh, and Egyptian) were mapped to the water buffalo reference genome of the Mediterranean breed (GCA_003121395.1, the UOA_WB_1 assembly). These mapped reads of RNA-seq data were first sorted and indexed using Samtools (Li et al., 2009; Li, 2011) along with the indexed reference genome (GCA_003121395.1). Then, coverage extraction of each nucleotide was performed using Samtools *mpileup*. Further, SNPs and InDels were extracted using bcftools (Danecek et al., 2021) *call*. Finally, significant SNPs were filtered using bcftools *view* at p-value <0.05, read depth >10, quality depth >30, minimum root mean square mapping >40, and flanking sequence length =50. This was followed by functional annotation of extracted SNPs and InDels using Perl script utilizing the annotation file of the genome of Mediterranean buffalo (GCA_003121395.1).

### Identification of SSRs Markers

MIcroSAtellite (MISA) (Beier et al., 2017) was used to extract SSRs from genome assemblies of all the five breeds utilizing parameters such as ≥10, ≥6, ≥5, ≥4, and ≥4 repeats for mono, di, tri, tetra, and penta nucleotide (nt) motifs, respectively along with length of compound SSRs ≤100 nt and minimum distance between two SSRs ≥50 nt (Zhao et al., 2017). The functional annotation of mined SSR markers was performed using Perl scripts utilizing the annotation of the Mediterranean buffalo RefSeq genome (GCA_003121395.1). Finally, based on the result of MISA, primer3 software (Untergasser et al., 2012) was used to design the primer pairs at default parameters, taking the flanking sequences of SSRs of the Mediterranean breed.

### Identification of microRNAs

For the prediction of miRNAs, first-known miRNAs and pre-miRNAs of *Bos tauras* from miRBase (Griffiths-Jones et al., 2006) were collected and duplicates were removed using CD-HIT (Huang et al., 2010). The pre-miRNA sequences of non-redundant *Bos tauras* miRNAs were aligned with the buffalo RefSeq genome (GCA_003121395.1) using BLASTn and sequences with 0 gap and ≤3 mismatches were taken along with 500 nt up and downstream stretches, making these >1000 nt length sequences (Altschul et al., 1990). Further, 200 nt fragments were taken from these sequences by using 25 nt sliding windows using the SegKit tool (Shen et al., 2016). The obtained sequences were again clustered using CD-HIT to obtain non-redundant sequences. Non-redundant sequences were used to predict the secondary structure by RNAfold (Lorenz et al., 2011) at minimum free energy (MFE) > −20. Further, sequences with <60 nt, non-AUGC, and multi-loop in structure, and pseudo pre-miRNAs were removed by Triplet-SVM classifier (Xue et al., 2005). These putative pre-miRNAs were used for further prediction of mature miRNAs using MiRdup (Leclercq et al., 2013). Finally, psRANTarget (Dai and Zhao, 2011) was used at an expectation value of 2 to predict mRNA targets of predicted miRNAs.

### Identification of Circular RNAs

For the identification of circRNAs from the mapped RNA-seq reads of 31 buffalo tissues, CIRI v2.0.4 (Gao et al., 2015) was used. As circRNA-looping sites cannot be aligned directly to the genome, find_circ (Memczak et al., 2013) was used for the first 20 base pairs of each read end that were incompatible with the genome to anchor independent reads, thus map them with the buffalo reference genome (GCA_003121395.1), and finally to find only the mapped site. If the two anchors aligned in the linear region were in the reverse direction, anchor reads were extended until circRNA junctions were found. The sequence was considered a circRNA if the two sides of sequences corresponded to GT/AG splicing signals as mentioned by Fu et al. (2018). CIRI was also used to annotate circRNAs by using the annotation file of the GCF_003121395.1 genome assembly.

### Identification of Long Non-Coding RNAs

For the identification of lncRNAs, from RNA-seq data of 31 buffalo tissues, first, mapping was performed using HISAT2 (Kim et al., 2019), followed by assembly using Stringtie v1.3.5 (Pertea et al., 2015). Then, putative lncRNAs were predicted from assembled reads using CPC2 (Kang et al., 2017) and passed through subsequent steps (a and b) for further validation as non-coding transcripts, i.e., 1) the transcripts with length ≥200bp, open reading frame (ORF) ≤100 aa, strand information (+/- strand), and CPC2 score <0.5 were selected using OrfPredictor (Min et al., 2005) and passed through annotation using the annotation file of the GCF_003121395.1 genome assembly by GffCompare (Burset and Guigo, 1996). 2) These were then searched against the NCBI-nr protein database through blastx (E value 0.01, coverage >80%, and identity >90%) and the Pfam protein database through HMMER (Finn et al., 2011). Finally, the validated lncRNAs were classified based on origin of lncRNAs as *i* (within a reference intron)*, j* (alternative lncRNAs isoforms of known genes)*, o* (lncRNAs with exonic overlap with a known transcript)*, u* (intergenic lncRNAs), and *x* (exonic overlap on the opposite stand) as classified by Roberts et al. (2011). Transcripts with FPKM ≥0.5 for multi-exon transcripts and FPKM ≥1 for single-exon transcripts were selected as lncRNAs.

### Development of Buffalo Web Genomic Resource, *BuffGR*

**FIGURE 1:**
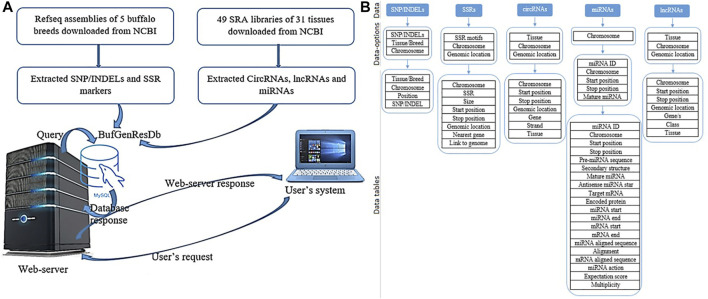
**(A)** Database preparation and data retrieval for *BuffGR*; **(B)** Layout of data, data options, and data tables of *BuffGR*.

The Buffalo Genomic Resource Database, *BuffGR* is a ‘*three tier architecture*’ relational database developed using client, server, and database tiers. The analyzed datasets were catalogued in *BuffGR* on a Linux server. The following steps were involved in the development of *BuffGR* (Figure 1A): 1) Extraction of SNPs/InDels, SSR markers, lncRNAs, miRNAs, and circRNAs from the reference genomes of different breeds of buffalo and SRA data of 31 tissues of buffalo. These data are absolute, rather than having relative quantification. 2) Development of relational database in MySQL version 10.4.17, which includes 11 tables for all the fields, *namely,* for SNPs/InDels, SSR markers, lncRNAs, miRNAs, and circRNAs (Figure 1B); 3) development of web interface in PHP, HTML, and Java. Web hosting of this interface was done by Apache2 server version 3.2.4. A request was sent to the web server from the user’s system in PHP. A query was generated following the request of the user on the web server and sent to MySQL. The database response was prepared in MySQL and sent back to the web server. Finally, a response prepared in PHP was displayed in the user’s system.

## Results

### Identification of SNPs and InDels

**FIGURE 2:**
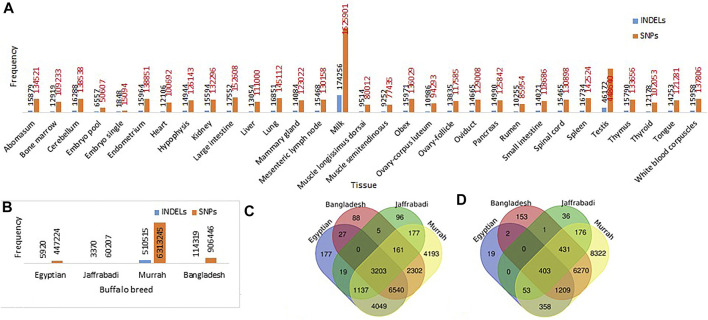
Frequencies of SNP/InDels in **(A)** 31 different buffalo tissues **(B)** different breeds of buffalo: Common and unique genes with abundance of **(C)** SNPs and **(D)** InDels in different breeds of buffalo.

A total of 6028881 SNPs and 613403 InDels were extracted from the set of 31 buffalo tissues. The highest number of SNPs and InDels was extracted from milk tissue (1625901 SNPs/174256 InDels) followed by testis (448640 SNPs/46172 InDels) and large intestine (152608 SNPs/17552 InDels) (Figure 2A). However, the variants detected breed-wise showed a maximum number of SNPs and InDels in the Murrah breed (6313245 SNPs/510515 InDels), followed by Bangladesh (906446 SNPs/114319 InDels) and Egyptian (447224 SNPs/5920 InDels), while the least was seen in Jaffarabadi (60207 SNPs/3370 InDels) (Figure 2B). Table 3 represents the extracted tissue-wise genes showing abundance of SNPs and InDels by functional annotation. A total of 7727122 SNPs and 634124 InDels were collectively distributed in the four breeds of buffalo (Murrah, Bangladesh, Jaffarabadi, and Egyptian) with reference to the Mediterranean breed. From functional annotation of breed-wise SNP/InDels, 12326/8469, 15152/2044, 4798/1100, and 21762/17222 genes were found to have abundance of SNPs/InDels in Bangladesh, Egyptian, Jaffrabadi, and Murrah breeds, respectively (Figure 2C for SNPs and Figure 2D for InDels). SNP discovery plays an important role in obtaining varying alleles associated with different traits of interest (Mishra et al., 2020). This can be useful in marker trait association studies for various traits (Pareek et al., 2008).

**TABLE 3:** Annotated genes with abundance of extracted SNP/InDels from buffalo tissues.

Tissue	Genes with SNPs	Genes with InDels	Tissue	Genes with SNPs	Genes with InDels
Tongue	14392	6944	Muscle longissimus dorsai	12381	5149
Rumen	13503	5751	Muscle semitendinosus	12428	5038
Obex	15038	7404	Small intestine	14322	6867
WBC	13422	6813	Large intestine	15438	7879
Testis	16121	8588	Ovary-corpus luteum	13538	5821
Thyroid	14227	6429	Ovary follicle	14208	6751
Heart	13279	6125	Cerebellum	14711	7385
Thymus	14602	7179	Endometrium	14882	7376
Oviduct	14728	7109	Mesenteric lymph node	14445	7189
Spleen	14629	7479	Mammary gland	14674	7052
Liver	13969	6593	Spinal cord	14583	7229
Pancreas	14620	7128	Bone marrow	13376	6252
Kidney	14726	7303	Embryo pool	9531	3510
Lung	14989	7600	Embryo single	6008	1338
Testis	16121	8588	Hypophysis	14763	7144
Milk	16090	9308	Abomasum	14877	7514

A total of 12 genes (SPP1: chr7, SCD: chr23, SREBF1: chr3, STAT1: chr2, TG: chr15, LALBA: chr4, INSIG2: chr2, GHRL: chr21, DGAT1: chr15, CSN1S1: chr7, BTN1A1: chr2, ADRA1A: chr3) with abundance of milk tissue SNPs from the present study were found to be common out of 19 candidate genes reported to be associated with milk production trait by Du et al. (2019) (Table 4). We also found 10 genes (COL1A2, APOB, GDF7, KLHL29, NRXN1, RGS22, VPS13B, MFSD14A, SLC35A3, PALMD) with abundance of SNPs of different breeds from the present study to be common out of 12 candidate genes for different QTL traits such as milk yield, fat yield, protein yield, fat %, and protein % identified from GWAS analysis of Italian Mediterranean buffalo using the SNP-ChIP technique by Iamartino et al. (2017) and Liu et al. (2017) (Table 4).

**TABLE 4:** Genes with abundance of extracted tissue/breed SNPs found to be common within the reported candidate genes of QTL traits.

Genes with abundance of SNPs: Chromosome (reported candidate genes)	Total SNPs (within respective genes)	Tissue/breed of extracted SNPs	QTL trait	Reference
SPP1: chr7, SCD: chr23, SREBF1: chr3, STAT1: chr2, TG: chr15, LALBA: chr4, INSIG2: chr2, GHRL: chr21, DGAT1: chr15, CSN1S1: chr7, BTN1A1: chr2, ADRA1A: chr3	15, 22, 17, 53, 122, 04, 18, 03, 19, 13, 05, 01	Milk tissue	Milk production	Du et al. (2019)
COL1A2: chr8, APOB: chr12	112, 193	Murrah, Bangladesh, Egyptian, Mediterranean	Milk yield	Iamartino et al. (2017)
GDF7: chr12	1598	Murrah, Bangladesh, Mediterranean	Milk yield	Iamartino et al. (2017)
KLHL29: chr12	1458	Murrah, Bangladesh, Egyptian, Jaffrabadi, Mediterranean	Milk yield	Iamartino et al. (2017)
RGS22: chr15, VPS13B: chr15	3249	Murrah, Bangladesh, Egyptian, Jaffrabadi, Mediterranean	Milk yield, fat yield, protein yield	Liu et al. (2017)
	344			
MFSD14A: chr6, SLC35A3: chr6, PALMD: chr6	60, 41, 215	Murrah, Bangladesh, Egyptian, Mediterranean	Fat %, protein %	Liu et al. (2017)

### Identification of SSR Markers

**FIGURE 3:**
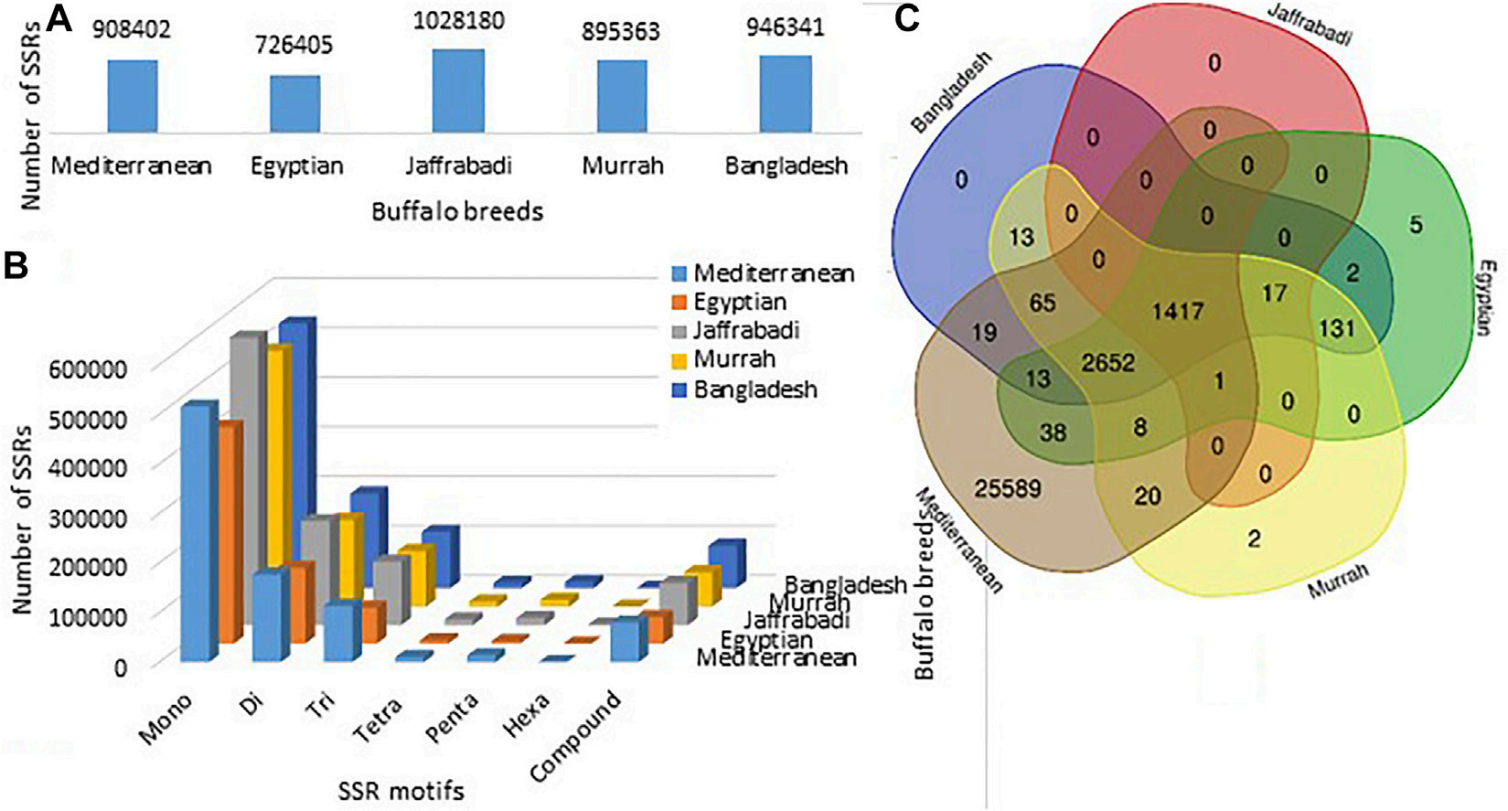
**(A)** Breed-wise frequencies of SSRs. **(B)** Breed-wise representation of different repeat motifs. **(C)** Common and unique genes with abundance of SSRs in the five breeds of buffalo.

Maximum number of SSRs were observed in Jaffrabadi (1028180), followed by Bangladesh (9463410) and Mediterranean (908402), while the least was found in Egyptian (726405) (Figure 3A). The number of SSRs based on repeat types (mono, di, tri, tetra, penta, hexa-nucleotide repeats) along with their proportions, frequency of SSRs per Mb, and distance between the two SSRs are listed in Table 5. In all the breeds, abundance of mononucleotides was observed which might be because of the inherent limitation of the chemistry employed in next-generation sequencing for data generation (Haseneyer et al., 2011) (Figure 3B). A similar higher proportion of mono repeats has been found in other animals like cattle, horse, and camel (Ma, 2016; Khalkhali-Evrigh et al., 2019). The relative distributions of various SSR motif lengths in genomes differ from species to species (Sharma et al., 2007). A total of 4329, 4284, 1435, 29822, and 4326 putative genes with an abundance of SSRs in Bangladesh, Egyptian, Jaffrabadi, Mediterranean, and Murrah breeds, respectively, were annotated. Figure 3C shows the common genes with abundance of SSRs in different breeds. The reported putative molecular markers can be used in marker trait association studies for buffalo genetic improvement programs (Sikka and Sethi, 2008; Bhuyan et al., 2010; Kannur et al., 2017).

**TABLE 5:** Breed-wise frequencies of SSRs, their proportions, SSR density, and distance between two SSRs in different repeat motifs.

Breeds	Repeats	Number	Proportion %	Frequency of SSRs per Mb	Distance between two SSRs in Kb
Mediterranean	Mono	515343	57.01	191.64	5.22
	Di	176276	19.24	65.55	15.25
	Tri	113425	12.40	42.18	23.71
	Tetra	10120	1.10	3.76	265.72
	Penta	13514	1.48	5.03	198.98
	Hexa	289	0.03	0.11	9304.68
	Compound	79435	8.75	29.54	33.85
Egyptian	Mono	436413	60.08	145.18	6.89
	Di	152723	21.02	50.81	19.68
	Tri	71979	9.91	23.95	41.76
	Tetra	6521	0.90	2.17	460.96
	Penta	5122	0.71	1.70	586.87
	Hexa	107	0.01	0.04	28092.99
	Compound	53541	7.37	17.81	56.14
Jaffrabadi	Mono	580010	56.41	154.26	6.48
	Di	209586	20.38	55.74	17.94
	Tri	127585	12.41	33.93	29.47
	Tetra	11688	1.14	3.11	321.70
	Penta	14154	1.38	3.76	265.65
	Hexa	343	0.03	0.09	10962.04
	Compound	84815	8.25	22.56	44.33
Murrah	Mono	516017	57.63	196.77	5.08
	Di	174481	19.49	66.53	15.03
	Tri	112283	12.54	42.82	23.36
	Tetra	10097	1.13	3.85	259.73
	Penta	13527	1.51	5.16	193.87
	Hexa	300	0.03	0.11	8741.53
	Compound	68658	7.67	26.18	38.20
Bangladesh	Mono	533868	56.41	192.71	5.19
	Di	190507	20.13	68.77	14.54
	Tri	113377	11.98	40.93	24.43
	Tetra	10575	1.12	3.82	261.96
	Penta	12246	1.29	4.42	226.22
	Hexa	268	0.03	0.10	10336.79
	Compound	85501	9.03	30.86	32.40

### Identification of microRNAs

**FIGURE 4:**
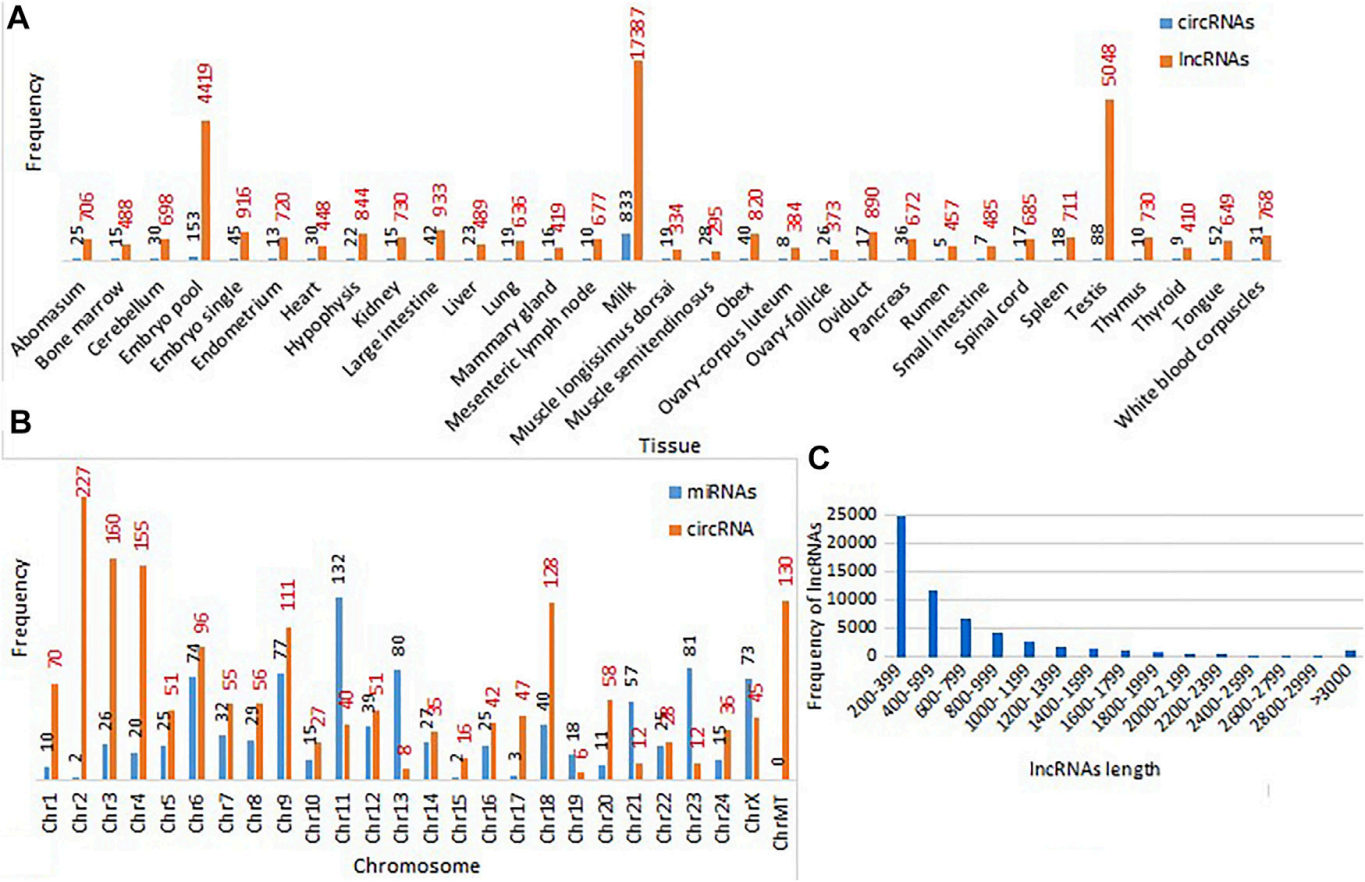
**(A)** Tissue-wise frequencies of circRNAs and lncRNAs **(B)** chromosome-wise frequencies of miRNAs and circRNAs; **(C)** length-wise frequencies of lncRNAs in buffalo.

We identified a total of 938 miRNAs from the genome assembly of the Mediterranean breed. The pre-miRNA sequences, secondary structure, target information, and location of origin were extracted for each miRNA along with mature miRNA sequence and anti-miRNA star sequence. It was observed that chromosome 11 had the maximum frequency of miRNAs (132 miRNAs) followed by chromosomes 23 (81 miRNAs) and 13 (80 miRNAs) (Figure 4B). A target search for 938 miRNAs was performed, out of which 88 miRNAs were found to have 3451 mRNA targets (predicted mode of action of miRNAs was cleavage of mRNA targets to destroy them or binding with mRNA targets to sequester them) and included in the web resource. Protein encoded by target mRNA, aligned as paired-unpaired sequences of the binding site between mRNA target and miRNA, were also mentioned in the web resource. The miRNAs have the future prospective to be used as biomarkers and for disease management and treatment. miRNAs can be used as a powerful tool to understand the regulatory mechanisms related to disease pathogenesis (Singh et al., 2020; Do et al., 2021).

### Identification of Circular RNAs

Out of the total 1702 circRNAs extracted from the 31 buffalo tissues, 1458 were unique circRNAs. Figure 4A shows that the maximum number of circRNAs was found in milk (833) tissues followed by embryo pool (153), testis (88), and tongue (52) tissues. Information of genomic localization into intron, exon, and intergenic regions of circRNAs along with genes of origin and strand of origin was extracted by functional annotation of circRNAs from different tissues which were catalogued in the web resource. The chromosome-wise distribution of circRNAs showed that most of the circRNAs originated from chromosome 2 (227), followed by chromosomes 3 (160) and 4 (155) (Figure 4B). circRNAs have multiple regulatory roles which can enrich breeding and improve economic traits related to buffalo (Fu et al., 2018; He et al., 2021; Yang et al., 2021).

### Identification of Long Non-Coding RNAs

A total of 44221 lncRNAs were identified in the 31 buffalo tissues. Abundance of lncRNAs was observed in milk tissue 17387) followed by testis (5048) and pooled embryo 4419) (Figure 4A). Genomic annotation based on the site of origin of lncRNAs found distribution of 37712 unique lncRNAs into five classes such as intron (14252), isoform/pseudogene (1308), exon (1358), intergenic (17134), and antisense exon (3659) regions. Protein and transcript information was also included for genic origin of lncRNAs. Genomic annotation of unique lncRNAs from all tissues depicted abundance in the intergenic (17134), followed by intron 14252) regions in our study. The graphical representation of lncRNA frequencies based on their length showed that most lncRNAs had a length of 200–399 bps and had a decreasing trend in frequency with increase in lncRNA length (Figure 4C). The role of lncRNAs in genomic studies has been found to be critical in linking the gap between livestock genotype and phenotype (Kosinska-Selbi et al., 2020).

### Development of Buffalo Web Genomic Resource

*BuffGR* is a comprehensive, first-of-its-kind web resource, with a holistic collection of buffalo molecular markers and variants of five buffalo breeds (Murrah, Mediterranean, Jaffarabadi, Bangladesh, and Egyptian). It is a user-friendly web resource, which catalogues SNPs, InDels, and SSRs along with ncRNAs such as mircoRNAs, lncRNAs, and circRNAs from the five buffalo breeds and 31 tissues. It has a left vertical section which provides access to varying sections of the web page including Home, Statistics, Data, and Team. The *Home* page includes the brief introduction of the buffalo web genomic resource along with a description about RNA/transcripts and molecular markers of buffalo. The *Statistics* section provides the statistics of extracted buffalo genomic data represented in the form of various graphs and pie charts.

**FIGURE 5:**
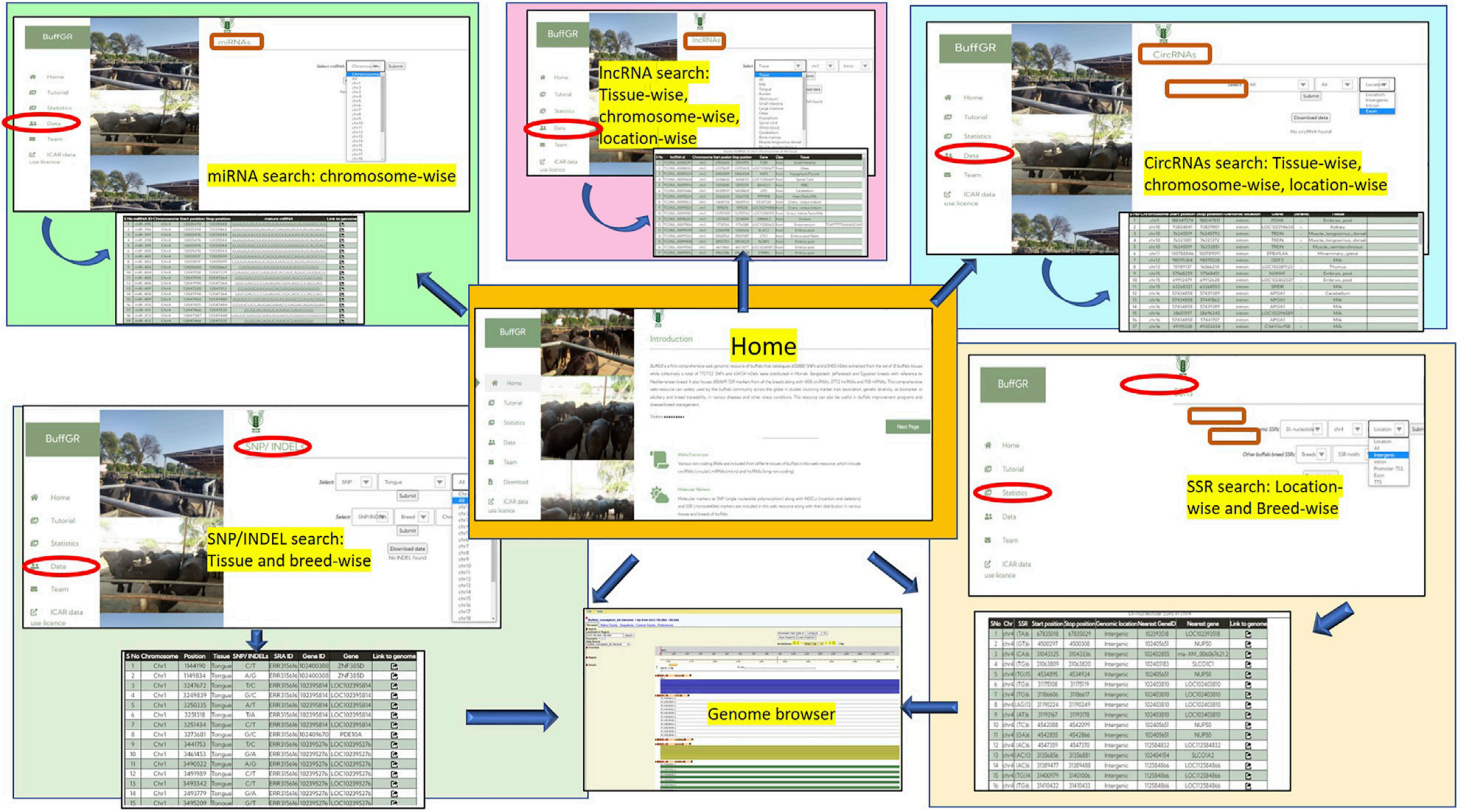
Web interface of BuffGR.

The *Data* section includes hyperlinked images of each data point included in the web resource, and by clicking on the image, the user navigates to the next page of the respective data which provides the user varying options including type of tissue or chromosome number or breed, etc. (as shown in detail in Figure 1B). After selecting the combination of options, the user gets a complete table of the related data. The last column of each table provides a hyperlink to the genome browser, which navigates to the genomic location of the respective marker or ncRNA. In the case of miRNAs, each miRNA sequence is hyperlinked, which navigates to its mRNA target/s wherever available; the *Team* page includes the name of the team members with their profile. The *Tutorial* page guides users regarding the use of this web genomic resource (Figure 5).

### Utility of Buffalo Web Genomic Resource

The computational approach of discovery of SSR markers, SNPs, and InDels along with miRNAs, lncRNAs, and circRNAs utilizing the available genomic data of different breeds resulted in a ready-to-use, user-friendly, rapid, and economical approach for genomic resource development. The developed web resource, *BuffGR* can be of immense use to the international buffalo research community, which can utilize the information of genomic attributes from five breeds from India (Murrah and Jaffrabadi), Italy (Mediterranean), Bangladesh (Bangladesh), and Egypt (Egyptian). The catalogued SNP/InDel markers from different breeds could be used to study genetic diversity among different breeds of buffalo (Camargo et al., 2015; Deng et al., 2016; El-Halawany et al., 2017; Iamartino et al., 2017; Liu et al., 2017; Dutta et al., 2020). Highly variable SSR markers extracted in the present study could be utilized to find genetic diversity (Barker et al., 1997; Zhang et al., 2020). The SSR markers from different breeds could be used to find polymorphic SSRs (Moore et al., 1995) and their utilization in the study of genetic diversity of respective breeds (Moioli et al., 2001; Merdan et al., 2019; Vohra et al., 2021; Ünal et al., 2021). We also extracted ∼270000 polymorphic SSRs in the Mediterranean buffalo breed with respect to Murrah, Bangladesh, Jaffrabadi, and Egyptian breeds. The species-specific genetic markers (SNP/InDels and SSRs) can also be used as biomarkers of species to be used in the meat industry to trace adulteration or trafficking/traceability (Kannur et al., 2017).

Two coding variants were detected in the ASIP gene by Dutta et al. (2020), one synonymous variant at chr14:19947421 and another non-synonymous variant at chr14:19947429. Dutta et al. (2020) also reported that the alternative allele at the synonymous variant was not observed in Murrah, Surti, or Mediterranean breeds. The potential of extracted SNPs from this study as biomarkers can be seen from the example that the Murrah and Mediterranean breeds in the present study only had one non-synonymous SNP at 19947429 in the ASIP gene on chr14 as reported by Dutta et al. (2020). Significant SNPs could be utilized to find candidate genes specific to a certain function. The variants and SSRs can also be utilized in GWAS (El–Halawany et al., 2017) and later in MTA (marker trait association) analysis and QTL analysis by interval mapping (Deng et al., 2016; Mishra et al., 2020). The present study also shows the potential utilization of extracted markers in marker trait association as few of the genes with abundance of extracted tissue/breed SNPs were found in common with the candidate genes of the few reported QTL traits determined from GWAS studies. We found 12 genes with abundance of milk tissue SNPs to be in common with candidate genes of milk trait, and 10 genes with abundance of SNPs from different breeds to be in common with candidate genes of QTL traits such as milk yield, fat yield, protein yield, fat %, and protein % from other GWAS analyses (Iamartino et al., 2017; Liu et al., 2017; Du et al., 2020).

Tissue-specific lncRNAs could be helpful in studying post-transcriptional regulation by targeting certain mRNAs by cleaving or binding (Zhang et al., 2021) with target mRNAs. lncRNAs could be competitors of miRNAs, which targeted certain mRNAs, where lncRNAs sequestered miRNAs by binding to them and preventing miRNA from cleaving the respective mRNA (Li et al., 2020). Also, tissue-specific lncRNAs could be helpful in utilization in transcriptional regulation by targeting or modulating transcription regulatory proteins by facilitating their binding to a certain site or blocking binding at their target site (Cai et al., 2019; Pan et al., 2021). Another important fact is that the provided tissue-wise lncRNAs are the largest reported group of annotated lncRNAs of buffalo in a single study while several studies report tissue-specific lncRNAs in various species of livestock such as *Bos taurus*, *Gallus gallus*, *Sus scrofa* (Kosinska-Selbi et al., 2020), and *Bos indicus* (Alexandre et al., 2020). The TCONS_00011978 lncRNA, identified from muscle tissue in the present study, was reported to have regulatory potential in muscle with the highest degree of connectivity within the muscle network by Alexandre et al. (2020), reaffirming the potential of our extracted lncRNAs to be utilized in various future studies of buffalo. The buffalo miRNAs and their target mRNAs extracted in the present study can be utilized in post-transcriptional regulation of certain mRNAs and their encoding proteins by cleaving or binding with their target mRNAs (MacFarlane and Murphy, 2010; Hammond, 2015; Chen et al., 2020; Singh et al., 2020) along with recognition, de-capping, and degradation of 3′ UTR, and de-adenylation and adenylation of 3’ UTR of mRNAs (Shukla et al., 2011). The miRNAs could be used to find their lncRNAs target; action of miRNAs on lncRNAs could be sequestering them by binding or destroying them by cleaving (Assmann et al., 2019; Xie et al., 2020). The tissue-wise extracted circRNAs in the present study could be utilized in the studies of tissue-specific post-transcriptional regulation involving circRNAs and their role in various buffalo diseases (Gao et al., 2018; He et al., 2021; Lei et al., 2021; Yang et al., 2021).

SNP and SSR markers can also be used in parentage and relatedness testing required in breeding and conservation programs (Labuschagne et al., 2015). SNP markers can also be used in estimating inbreeding and effective population sizes required in conservation management monitoring genetic diversity (Panetta et al., 2017). They can be used to compute global co-ancestries of un-pedigreed populations. Such an approach can be of immense use in formulation of selective mating plans based on minimum co-ancestry mating and minimizing inbreeding (Fernández et al., 2005). Both SSR and SNP markers can be used in individual animal identification and breed traceability (Zhao et al., 2020). Water buffalo miRNAs and SNPs can be further used as genomic resources. Such use has been reported in cattle where SNPs and miRNAs have been found associated with bovine phenotypes to be used in breed improvement (Sousa et al., 2021).

## Conclusion

Through this study, we report the first comprehensive and user-friendly web genomic resource for buffalo (*BuffGR*) including genomic findings of five commercially important buffalo breeds, namely Mediterranean, Egyptian, Bangladesh, Jaffrarabadi, and Murrah. BuffGR catalogues a total of 6028881 SNPs and 613403 InDeLs extracted from the set of 31 buffalo tissues. Collectively, a total of 7727122 SNPs and 634124 InDels were distributed in the four breeds of buffalo (Murrah, Bangladesh, Jaffarabadi, and Egyptian) with reference to the Mediterranean breed. The web resource has 4504691 SSR markers from all the breeds, 1458 unique circRNAs and 37712 lncRNAs from 31 buffalo tissues, and 938 miRNAs from the genome assembly of the Mediterranean breed. This information can be widely used by the buffalo researchers across the globe for studying the genetic diversity among the different breeds of buffalo, studies involving post-transcriptional regulation, and their role in various buffalo diseases. The provided markers can be used as biomarkers in the meat industry to trace adulteration, trafficking, and breed traceability. These can be used not only for knowledge discovery research but also for marker trait association, which will be helpful in the improvement and management of buffalo breeds.

## Data Availability

The datasets presented in this study can be found in online repositories. The names of the repository/repositories and accession number(s) can be found in the article/Supplementary Material.
